# Beyond zirconia: hafnia redefines support design for In_2_O_3_-based catalyst in CO₂-to-methanol synthesis

**DOI:** 10.1093/nsr/nwag242

**Published:** 2026-04-22

**Authors:** Zhiqiang Rao, Wei Zhou, Ding Ma

**Affiliations:** Beijing National Laboratory for Molecular Sciences, New Cornerstone Science Laboratory, College of Chemistry and Molecular Engineering, Peking University, China; Beijing National Laboratory for Molecular Sciences, New Cornerstone Science Laboratory, College of Chemistry and Molecular Engineering, Peking University, China; Beijing National Laboratory for Molecular Sciences, New Cornerstone Science Laboratory, College of Chemistry and Molecular Engineering, Peking University, China

Carbon dioxide (CO_2_) hydrogenation to methanol using renewable H_2_, known as green methanol synthesis, offers a compelling route to mitigate carbon emissions while enabling sustainable chemical manufacturing. For decades, Cu/ZnO/Al_2_O_3_ has been the industrial benchmark for methanol synthesis and the most extensively studied catalyst for CO_2_ hydrogenation. Yet under CO_2_-rich conditions, its intrinsic limitations become evident. Methanol selectivity is constrained by the competing reverse water–gas shift (RWGS) reaction, while the water generated during the reaction accelerates catalyst restructuring, eventually leading to severe deactivation [1]. These challenges have motivated the exploration of alternative catalysts with improved methanol selectivity and stability. In this context, In_2_O_3_ has emerged as a particularly promising candidate. Early density functional theory studies in 2013 identified oxygen vacancies on the In_2_O_3_(110) surface as key active sites that favor methanol formation [2]. That prediction is subsequently validated experimentally in 2015 [3], when methanol synthesis over activated In_2_O_3_ was directly demonstrated. The significance of this system was further established in 2016 by Javier Pérez-Ramírez’s team. They reported an In_2_O_3_ catalyst supported on monoclinic zirconia (*m*-ZrO_2_), exhibiting high activity, near-complete methanol selectivity and remarkable long-term stability under industrially relevant conditions [4].

**Figure 1.**
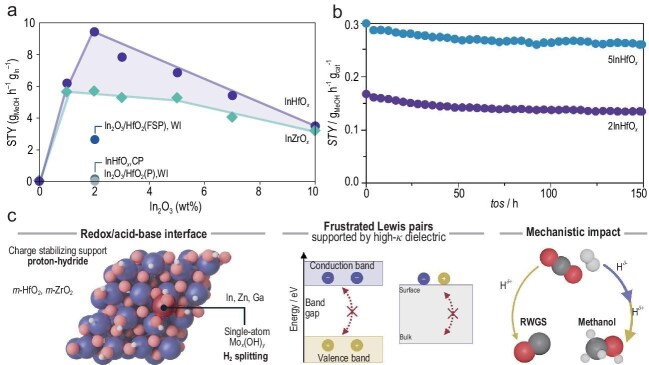
(a) Space-time yield of methanol over InHfO_*x*_ and InZrO_*x*_ catalysts with varying In_2_O_3_ content. (b) Stability test over selected InHfO_*x*_ catalysts. Reaction conditions: *T* = 553 K, *P* = 50 bar, H_2_/CO_2_ = 4 and gas hourly space velocity (GHSV) = 24 000 cm^3^ h^−1^ g_cat_^−1^. (c) Design principles for high-κ dielectric catalysts for efficient methanol synthesis from CO_2_ hydrogenation. Reproduced with permission from ref. [5].

Despite these advances, a key question has remained unresolved: Why is *m*-ZrO_2_ such an effective support, and can it be further surpassed? In a recent article, Javier Pérez-Ramírez and colleagues provide a compelling answer by demonstrating that monoclinic hafnia (*m*-HfO_2_) can outperform *m*-ZrO_2_ [5]. More importantly, they reveal that the support is not merely a structural scaffold, but an active component of the catalytic interface. Nanostructured indium–hafnium oxides (InHfO_*x*_), synthesized by flame spray pyrolysis, deliver up to 70% higher indium-specific methanol productivity than the benchmark indium–zirconium oxide (InZrO_*x*_) catalyst (Fig. 1). The optimal catalyst, containing 2 wt% In_2_O_3_, achieves a methanol productivity of 9.3 g_MeOH_ h^−1^ g_In_^−1^, while maintaining stable operation over 150 h.

The significance of this work lies not only in its performance gains but also in the design principles it establishes. The authors propose that an ideal support for the In-based catalyst in CO_2_-to-methanol synthesis should combine three features: a stable monoclinic lattice, a wide bandgap that can stabilize charged hydrogen species and the ability to stabilize low-valence indium species associated with oxygen vacancies. Hafnia emerged as a unique candidate that satisfies all three criteria. Structurally, HfO_2_ is analogous to ZrO_2_, preserving the favorable geometric features of the benchmark catalyst. Electronically, however, it is distinct, creating a modified interfacial environment that more effectively stabilizes single-atom InO_*x*_ as reactive species than zirconia. This insight elevates the work beyond a simple material replacement, instead presenting a strategy for catalyst design guided by mechanistic insight.

Further insights are obtained from the analysis of the catalysts’ surface chemistry. Catalysts prepared via conventional routes, such as wet impregnation and coprecipitation, display negligible activity despite possessing the same monoclinic hafnia phase. In contrast, a hydrothermally prepared hydroxylated HfO_2_ support restores activity close to that of the flame-made system, confirming that surface hydroxylation rather than the synthesis method itself is crucial to achieving high performance. Importantly, the most effective active motif is not InO_*x*_ patches or clusters, but atomically dispersed indium anchored on hydroxylated HfO_2_, which effectively enhances the indium utilization. The authors further suggest that the wide band gap and high dielectric constant of HfO₂ facilitate the stabilization of cooperative hydride–proton species at the surface while suppressing electron delocalization into the bulk, thereby promoting the methanol pathway over the competing RWGS reaction (Fig. 1).

Taken together, this study highlights a paradigm shift in catalyst design. It demonstrates that catalyst supports can be deliberately engineered to regulate metal dispersion, vacancy structure, hydrogen speciation and charge localization in a concerted manner. By addressing a long-standing challenge in CO₂-to-methanol catalysis, this work opens new avenues for the rational design of oxide-supported catalysts. Although the practical deployment of hafnia may be constrained by its relative scarcity, the insights gained here are broadly applicable. More importantly, the study underscores that materials traditionally considered inert support can become transformative when their electronic and chemical roles are fully exploited.
